# CD98-induced CD147 signaling stabilizes the Foxp3 protein to maintain tissue homeostasis

**DOI:** 10.1038/s41423-021-00785-7

**Published:** 2021-11-10

**Authors:** JieJie Geng, Ruo Chen, Feng-fan Yang, Peng Lin, Yu-meng Zhu, Xianghui Fu, Ke Wang, Zhuan Feng, Jiao Wu, Hai Zhang, Qi-jing Li, Zhi-Nan Chen, Ping Zhu

**Affiliations:** 1 Department of Clinical Immunology, Xijing Hospital, Fourth Military Medical University, Xi’an, P. R. China; 2 National Translational Science Center for Molecular Medicine & Department of Cell Biology, Fourth Military Medical University, Xi’an, P. R. China; 3 Department of Immunology, Duke University Medical Center, Durham, NC USA

**Keywords:** Treg, Stability, CD147, CD98, CDK2, Immune therapy, Lymphocytes, Autoimmunity

## Abstract

Regulatory T cell (Treg) stability is necessary for the proper control of immune activity and tissue homeostasis. However, it remains unclear whether Treg stability must be continually reinforced or is established during development under physiological conditions. Foxp3 has been characterized as a central mediator of the genetic program that governs Treg stability. Here, we demonstrate that to maintain Foxp3 protein expression, Tregs require cell-to-cell contact, which is mediated by the CD147-CD98 interaction. As Tregs are produced, CD147, which is expressed on their surface, is stimulated by CD98, which is widely expressed in the physiological environment. As a result, CD147’s intracellular domain binds to CDK2 and retains it near the membrane, leading to Foxp3 dephosphorylation and the prevention of Foxp3 degradation. In addition, the optimal distribution of Foxp3+ Tregs under both pathological and physiological conditions depends on CD98 expression. Thus, our study provides direct evidence that Foxp3-dependent Treg stability is reinforced in the periphery by the interaction between CD147 and CD98 in the surrounding environment. More importantly, Tregs with high CD147 expression effectively inhibit inflammatory responses and maintain Foxp3 stability, which has guiding significance for the application of Tregs in immunotherapy.

## Introduction

Regulatory T cells (Tregs) are essential for immune tolerance and the maintenance of immune homeostasis [1]. Although fewer than 10% of circulating CD4 + T cells are Tregs, Tregs play an essential role in preventing antigen-induced immunity and limiting excessive inflammation in a variety of animal models and human diseases, including immune-dysregulation polyendocrinopathy enteropathy X-linked syndrome, type 1 diabetes, rheumatoid arthritis, and cancer [2–4]. In particular, many studies have investigated the role of Tregs in inflammatory bowel disease (IBD). In IBD patients, increased numbers of Tregs are found in the lamina propria and mesenteric lymph nodes [5–7], suggesting that Tregs may be recruited to and expanded in the intestinal mucosa in the context of an inflammatory environment. Treg therapy in preclinical animal IBD models has shown marked potential in the treatment of immune-mediated diseases and has laid the foundation for its applications in human therapy. Adoptive transfer was used in most cases, but there is no guarantee that the transplanted Tregs will remain stable for a long time. Therefore, new protocols need to be developed to isolate and use specific Tregs that achieve their optimal clinical application.

The inflammatory environment imposes a unique challenge on Treg cells. Similar to any other type of T cell, Tregs need to be immersed in such an environment to perform their functions and lack the ability to exert their effects over long distances. Moreover, inflammatory stimulation elicits multiple destabilization mechanisms to decrease the expression of Foxp3, a Treg lineage-specifying transcription factor that is crucial for the development, function, and stability of Tregs [8]. However, at present, it has become apparent that Foxp3 transcription alone does not adequately define Treg stability, as there is increasing evidence that many factors can affect Foxp3 protein stability. In response to inflammatory cytokines, such as IL-6, Tregs degrade Foxp3, which is mediated by increased polyubiquitination by STUB1 and decreased deubiquitination by USP-7 [9, 10]. In addition, there is a strict balance between Foxp3 polyubiquitination and lysine acetylation, and this is another key mechanism that regulates Foxp3 stabilization [11]. Phosphorylation is an additional modification that affects the Foxp3 protein. Veerle Fleskens et al. discovered that Nemo-like kinase directly phosphorylates Foxp3 to stabilize protein expression [12], whereas cyclin-dependent kinase 2 (CDK2)-mediated phosphorylation of Foxp3 reduces protein stability [13], suggesting that specific phosphorylation events exert distinct effects on Foxp3 stability. In summary, multiple mechanisms, including posttranslational modifications and the activity of various regulatory factors, regulate the protein levels of Foxp3 in a cell. However, these are all intracellular regulatory factors, and there is currently little knowledge concerning environmental factors that might function to regulate Foxp3 stability.

CD147 is a glycosylated cell surface protein important for vision, spermatogenesis, and other physiological phenomena, and it plays significant roles in the pathogenesis of various diseases, including cancer [14]. CD147 contains two Ig domains in its extracellular segments and a conserved Glu in its transmembrane domain, suggesting that it may interact with other proteins to execute its functions [15]. Indeed, CD147 binds some monocarboxylate transporters (MCTs), assisting with their localization to cell membranes where they perform their transport functions, and this function is essential for vision formation [16]. CD147 also interacts with CD98, mediating its homotypic aggregation to attenuate the CyPA-induced chemotactic effect in Jurkat T cells [17]. CD147 is a receptor of cyclophilins, which mediate the migration of inflammatory leukocytes [18], and the receptor for the invasive proteins RH5 and RAP2 [19, 20], which mediate the invasion of plasmodium. In summary, a variety of proteins are known to bind distinct sites on CD147 to execute their divergent functions.

Importantly, CD147 is expressed on the surface of Tregs, and its presence can divide CD4 + CD25 + cells into distinct subsets. Phenotypic and functional analyses suggest that CD147 expression indicates the switch between resting CD45RA + and activated CD45RO + subsets within the Foxp3 + T cell population, thereby distinguishing an activated and highly suppressive CD45RO + Treg subset. Based on the expression levels of CD147, Foxp3+ cells can be divided into three groups. Interestingly, Tregs with higher CD147 expression also express more CTLA-4 and IL-10 but produce low levels of cytokines, such as IL-2, TNFα, IFNγ, and IL-17, suggesting that Tregs with high CD147 expression are more stable [21]. However, although CD147 is a known marker of activated Tregs, its underlying mechanisms remain unclear.

In this study, we show that individual Tregs are not stable and that CD147 is an important extracellular molecule that regulates the function and stability of Tregs by regulating Foxp3 protein stability. CD147, which present on the Treg surface, is stimulated by CD98, which is widely expressed in the physiological environment, to maintain Foxp3 protein stability. More importantly, Tregs with high CD147 expression can effectively inhibit inflammatory responses and maintain Foxp3 stability, which has guiding significance for the application of Tregs in immunotherapy.

## Materials and methods

### Mice

Studies of CD147^T-KO^ mice on the C57Bl/6 background have been previously published [22]. Wild-type (WT) female C57BL/6 mice were purchased from the Fourth Military Medical University, Laboratory Animal Center, and white C57BL/6 mice were obtained from the Biomedical Analysis Center of the Third Military Medical University. Rag1-/- mice were purchased from Jackson Laboratories. NOG (NOD/Shi-scid/IL-2Rγnull) mice were purchased from Beijing Vitalstar Biotechnology. All the mice were housed under specific pathogen-free conditions. The mice were treated according to animal protocols that were approved by the Institutional Animal Care and Use Committee.

### FACS, intracellular staining, and flow cytometry analysis

Cell sorting was performed using a MoFlo legacy sorter, and the purity of the sorted cells was determined postsorting to be at least 98% for each experiment. Cell analysis was performed using BD Fortessa. Foxp3 staining was performed using a Foxp3/Transcription Factor Staining Buffer Set from eBioscience with an anti-mouse Foxp3 antibody (clone MF-14) and anti-human Foxp3 antibody (clone 259D) following the suggested protocol. For intracellular cytokine staining, cells were stimulated with 0.9 nM PdBU and 0.5 μg/mL ionomycin (Sigma–Aldrich) in the presence of 5 μg/mL brefeldin A and 2 μM monensin (eBioscience) for 4 h. The cells were treated with a Fixation/Permeabilization solution kit from BD Cytofix/Cytoperm^™^ and stained with anti-cytokine antibodies. Fluorochrome-conjugated antibodies recognizing CD4, CD8, CD25, CD69, CCR7, CD62L, CD45RA, IL-17, IFN-γ, TNF-α, CD45.1, and CD45.2 were purchased from BioLegend.

### Adoptive transfer and inflammatory bowel disease model

For the IBD model, 2.5 × 10^4^ CD45.2 + CD147-sufficient or CD45.2 + CD147-deficient Tregs were sorted from the lymph nodes and spleens of CD147f/f Lck–Cre mice or their WT littermate controls and mixed with 5 × 10^5^ CD4 + CD25 − conventional T cells from WT CD45.1 + B6 mice. The cell mixtures were then intraperitoneally transferred into Rag1-/- mice. The recipient mice were monitored closely for changes in body weight for 3–4 months and then euthanized for immunological and histological analysis.

### Murine/human Treg and naïve T cell isolation

Peripheral blood mononuclear cells were isolated from the peripheral blood of mice or humans using a Ficoll-Paque PLUS gradient (GE Healthcare). Then, the cells were labeled with antibodies against CD4, CD25, CD62L and CD45RA (Biolegend). Human ex vivo Tregs and naïve T conventional (nTconv) cells were defined as CD4 + CD25 + cells and CD4 + CD25-CD62L + CD45RA + cells, respectively. Murine ex vivo Tregs and nTconv cells were defined as CD4 + CD25 + cells and CD4 + CD25- cells, respectively.

### In vitro iTreg induction

Human or murine ex vivo Tregs were cultured with anti-CD3/anti-CD28-coated microbeads (Miltenyi Biotec) at a 1:1 ratio and 50 IU/ml IL-2 (Peprotech) with or without 50 ng/ml IL-6 (Peprotech). To induce iTreg differentiation, nTconv cells were cultured with anti-CD3/anti-CD28-coated microbeads at a 1:1 ratio with 25 IU/ml IL-2, 10 µg/ml anti-IL6 and 10 ng/ml TGF-β.

### Immunoaffinity chromatography and mass spectrometry

Immunoaffinity chromatography was performed using an Affi-Gel Hz Immunoaffinity Kit (Bio–Rad Laboratories, Hercules, CA). Silver staining of sodium dodecyl sulfate polyacrylamide gels was performed using a Silver Stain for Mass Spectrometry Kit (Pierce). Trypsin-digested protein samples were analyzed using LTQ-Orbitrap XL (Thermo Fisher Scientific) mass spectrometry.

### Expression and purification of proteins

For the expression and purification of the extracellular portion of CD147 (ECD), cDNA encoding amino acids 22–205 of CD147 was inserted into pET21a (+) (Novagen) with NdeI and XhoI, with a C-terminal 6X His-tag. This construct was transformed into the *Escherichia coli* strain OrigamiB (DE3) and grown in LB medium, yielding a soluble CD147 extracellular domain. The bacterial pellet was resuspended and sonicated in 20 mm Tris-HCl, pH 8, and centrifuged at 14000 *g* for 30 min. The supernatant was applied directly to a HisTrap HP column, followed by a HisTrap Q ion exchange column (GE Healthcare). A Superdex 75 gel-filtration column (GE Healthcare) with PBS were used for the final purification step. For expression and purification of the intracellular portion of CD147 (ICD, 230-269) and the extracellular portion of CD98 (212-630 of CD98hc), the proteins were expressed and purified in the same way as for CD147.

### Surface plasmon resonance

SPR studies were performed using a Biacore T200 instrument. CD147ECD peptides were diluted in 10 mM sodium acetate (pH 4.5) and immobilized on a CM5 chip (Biacore, GE Healthcare). Purified proteins (as analytes) were injected over the chip at 10 µL/min. Bovine serum albumin (BSA) protein was used as the control protein, and the BSA signal was subtracted during the analysis of the kinetic parameters by ProteOn Manager Version 2.0 software (BioRad Laboratories).

For SPR interruption experiments, CD147 protein was incubated with the Fab fragment of 5A12 to form a CD147-5A12Fab complex, which was then isolated by size exclusion chromatography. The purified CD147-5A12Fab complex was injected onto chips to which the BSA, CD98, and caveolin-1 proteins were separately immobilized. Interactions between peptides and proteins were assessed using a Biacore T200 system (GE Healthcare Life Sciences) with a CM5 chip.

For the CD147ICD-CDK2 interaction, CD147ICD was immobilized on the CM5 chip surface, and CDK2 proteins (purchased from Sino Biological Inc.) were injected over the chip at 30 µL/min. Bovine serum albumin (BSA) protein was used as the control protein, and the BSA signal was subtracted during the analysis of the kinetic parameters by ProteOn Manager Version 2.0 software (Bio–Rad Laboratories).

### Coimmunoprecipitation

Coimmunoprecipitation (Co-IP) experiments were performed using a Pierce Co-IP Kit (Thermo Fisher Scientific). The following antibodies were developed in our laboratory and used for these experiments: 5A12 and 6H8.

### Histology

Tissues were excised, fixed in 4% (vol/vol) paraformaldehyde overnight at room temperature, and then embedded in paraffin before H&E staining. The histological scores of the colon in the IBD model were assessed as previously described [23] in a double-blinded manner. In short, the results represent the sum of four scores: the percent area of involvement (scale of 0–4), severity of inflammation (scale of 0–4), extent of inflammation (scale of 0–4), and damage to crypts (scale of 0–4). Therefore, the final score ranges from 0 to 16.

### RNA-seq

RNA was isolated using MyOne Silane Dynabeads (Thermo Fisher Scientific) and then fragmented and barcoded using eight-base pair barcodes together with standard Illumina adaptors. The primers were removed using Agencourt AMPure XP bead cleanup (Beckman Coulter/Agencourt), and the samples were amplified with 14 PCR cycles. Libraries were gel purified and quantified using a Qubit high-sensitivity DNA kit (Invitrogen), and library quality was assessed using TapeStation high-sensitivity DNA tapes (Agilent Technologies). RNA-seq reactions were sequenced on an Illumina NextSeq sequencer (Illumina) according to the manufacturer’s instructions, sequencing 50-base pair reads. Analysis was performed using the CLC Genomics Workbench v.8.0.1 RNA-seq analysis software package (Qiagen). Briefly, reads were aligned (mismatch cost = 2, insertion cost = 3, deletion cost = 3, length fraction = 0.8, similarity fraction = 0.8) to the mouse genome, and differential expression analysis was performed (total count filter cutoff = 5.0). The results were normalized to reads per million. Gene-e (Broad Institute) was used to generate heat maps. Datasets generated during the current study are available on the Gene Expression Omnibus (GSE134153) and are also available from the corresponding author upon reasonable request.

### T cell-driven experimental colitis in humanized mice

For the IBD model, human CD4 + T cells were isolated from a healthy donor, and naïve T cells were sorted from the same person and stimulated with anti-CD3/CD28 magnetic beads in the presence of 50 U/ml IL-2 and 10 ng/ml TGF-β with or without 5A12 antibody. Five days later, 2 × 10^6^ induced Tregs were mixed with 1 × 10^6^ CD4 T cells.

Six- to eight-week-old NOG (NOD/Shi-scid/IL-2Rγnull) mice were divided into three groups and then intraperitoneally injected with 2 × 10^6^ human CD4 + T cells mixed with 1 × 10^6^ iTregs, CD147^low^ iTregs or CD147^high^ iTregs. Two weeks later, the proportion of hCD4 cells in mouse peripheral blood was ~10%. The mice were first sensitized by applying 150 microliters of a 2.5% TNBS (Sigma–Aldrich) solution in 50% ethanol to a 1 cm^2^ patch of bare skin at the base of the body. Seven days later, the mice were anesthetized via a single intraperitoneal injection of 0.1% Nembutal. The mice were given a single enema containing 0.25 mg of TNBS in a mixture of 50% ethanol and 50% phosphate buffered saline (PBS) in a volume of 50 microliters. All the animals were weighed daily, and colitis was assessed every 3 days after the rectal challenge with TNBS. For 5A12 antibody-treated mice, 10 mg/kg 5A12 dissolved in PBS was intraperitoneally administered starting at the same time as the injection of mixed human T cells, and was then, this antibody was administered every 2 weeks thereafter.

### Computational simulation of the CD147-CD98 complex

The structural model of the CD147-CD98 complex was generated by in silico docking using ClusPro (https://cluspro.bu.edu/home.php), a protein-protein docking server, as published in Nature Protocol. In detail, the X-ray structure files of CD98 (PDB ID: 2DH2), CD147 (PDB ID: 3B5H) and 5A12 (PDB ID 5H90) were obtained from the PDB and uploaded to ClusPro, where rigid body docking, clustering and energy minimization were automatically conducted. The top 10 outputs of cluster centers were filtered using two criteria revealed by our experimental data. The eligible model was used to analyze the key amino acid residues involved in the interaction and verified by subsequent mutation experiments.

### Multiplex fluorescent IHC

Multiplex immunofluorescence analyses were performed using 3 μm sections of formalin-fixed paraffin-embedded tissues. Slides were deparaffinized in xylene and hydrated using a series of decreasing ethanol solutions. After heat-induced antigen retrieval in either citrate (pH = 6) or TRIS-EDTA (pH = 9) buffer, samples were permeabilized with 0.5% Triton X-100, blocked with 5% goat serum-phosphate-buffered saline (PBS) and sequentially costained with antibodies recognizing CD98, CD147, CD4, Foxp3, IFNγ, IL17a, and DAPI. A TSA indirect kit (PerkinElmer) was used according to the manufacturer’s instructions. Slides were then microwaved to remove the primary and secondary antibodies, washed, and blocked again using blocking solution. The second primary antibody was applied, and the process was repeated, except that DAPI was applied rather than another primary antibody. After the unbound DAPI was washed off, the slides were coverslipped. In addition to the multiplex assay described above, a single color slide was generated for each antibody. Image analysis was performed using HALO software. The staining panels are as follows:

**Table:** 

Position	Antibody	Company	Dilution	Incubation	TSA dyes
1	IL-17	Abcam	1:200	60 min	650
2	IFNγ	Abcam	1:200	60 min	620
3	FoxP3	Abcam	1:500	60 min	690
4	CD147	Produced by our lab	1:1000	60 min	540
5	CD4	Abcam	1:100	60 min	520
6	DAPI	Perkin Elmer Opal 7-color kit	2 drops/ml	10 min

**Table:** 

Position	Antibody	Clone (host)/Company	Dilution	Incubation	TSA dyes
1	CD98	Abcam	1:1000	60 min	620
2	CD4	Abcam	1:100	60 min	690
3	CD147	Produced by our lab	1:1000	60 min	520
4	Foxp3	Abcam	1:500	60 min	540
5	DAPI	Perkin Elmer Opal 7-color kit	2 drops/ml	10 min	

### Statistical analysis

Statistical analyses were performed using GraphPad Prism software, version 5.0. Significant differences in the cell and mouse tests were analyzed using paired *t*-tests or unpaired *t*-tests with a two-tailed distribution. *p* < 0.05 was considered statistically significant. All multiplex fluorescent IHC images were analyzed using HALO software, and significant differences were analyzed by unpaired *t*-tests. Correlation analysis was performed by Pearson analysis. All the experiments presented in this study yielded reproducible results from a minimum of three independent replicates.

## Results

### Loss of CD147 expression destabilizes Tregs in the periphery

**Fig. 1:**
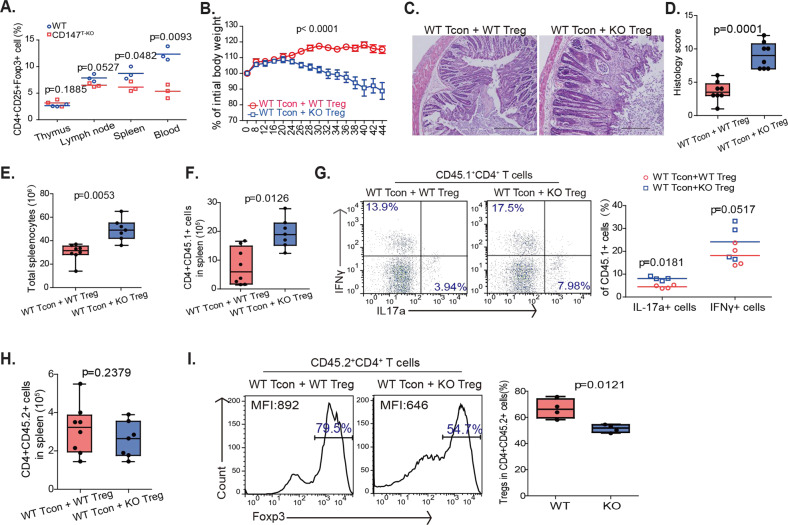
Loss of CD147 expression destabilizes Tregs in the periphery. **A** Comparison of Foxp3+ Treg numbers in the thymus, lymph nodes, spleen, and circulating blood of WT and CD147^T-KO^ mice by flow cytometry. **B–I** For the inflammatory bowel disease (IBD) model, 2.5 × 10^4^ CD45.2 + CD4 + CD25 + Tregs were sorted from the spleens of CD147^T-KO^ mice (*n* = 7) or their WT littermate controls (*n* = 8) and then mixed with 5 × 10^5^ CD4 + CD25 − conventional T cells from WT CD45.1 + B6 mice. Cell mixtures were intraperitoneally transferred into Rag1−/− mice. Recipient mice were euthanized 44 days later, and colon and spleen tissues were isolated for histopathological analysis. **B** Weight changes in recipient mice after adoptive transfer were normalized to their initial body weights before transfer. **C** Representative H&E stained images of colon tissues. **D** Summary of histopathological scores. **E** Absolute numbers of splenocytes. **F** Absolute numbers of CD4 + CD45.1+ cells in spleens. **G** CD45.1 + T cells from spleens were stimulated with PdBU and ionomycin for 4 h and then stained for the intracellular cytokines IFNγ and IL17a. **H** Absolute numbers of CD4 + CD45.2+ cells in spleens. **I** Percentages of Foxp3+ cells among CD45.2+ cells and mean fluorescence intensity (MFI) of Foxp3 staining in Foxp3+ Tregs

Previously, we reported that the early deletion of CD147 expression in thymocytes (CD147^T-KO^ mice [24]) leads to the enrichment of lymphocytes with innate features [22]. Moreover, in these CD147^T-KO^ mice, while Treg development in the thymus appeared to be normal, the ratio of Foxp3+ Tregs was reduced in peripheral organs, including lymph nodes, spleen, and circulating blood (Fig. S1A & Fig. 1A). This reduction was observed in both thymic-derived (Foxp3+Helios + ) and peripheral-converted (Foxp3+Helios−) [25] Treg cells (Fig. S1B & C). Moreover, this reduction is unlikely to be caused by defects in Treg thymic egress, since the surface expression of CD69 [23] (Fig. S1D), CCR7 [26] (Fig. S1E), and S1P1 [27] (Fig. S1F) was normal. Thus, we speculated that the reduction in Treg numbers observed in CD147^T-KO^ mice may be a postdevelopment event occurring in the periphery.

To investigate whether this reduction in peripheral Treg numbers in CD147^T-KO^ mice is Treg-intrinsic, we implemented a classic T-cell adoptive cotransfer approach in which naïve CD4 + conventional T cells (Tcon) and Tregs were cotransferred into lymphopenic hosts. At an effector:Treg ratio of 20:1, Tregs can barely maintain immune homeostasis, and any defect in Tregs may lead to the manifestation of colitis [28]. CD45.2 + CD4 + CD25 + Tregs were sorted from CD147^T-KO^ mice or WT mice and analyzed to ensure similar Foxp3 expression (Fig. S1G). Then, the cells were mixed with CD45.1+ naïve CD4 + Tcon cells from WT mice, and cotransfected into RAG1−/− recipients (Fig. S1H). As assessed by both weight change and pathological analysis, while WT Tregs were capable of mitigating colitis in recipients, mice receiving CD147-deficient Tregs developed colitis symptoms, including weight loss (Fig. 1B), massive leukocyte infiltration, and severe mucosal tissue damage in the colon (Fig. 1C, D). Accompanying this colon pathology, the numbers of total splenocytes (Fig. 1E) and, specifically, CD4 + effector T cells differentiated from transferred CD45.1+ Tcons, were significantly elevated (Fig. 1F). These effector T cells were also more inflammatory, as evidenced by their improved Th17 differentiation [29] (Fig. 1G). We also recovered the transferred Tregs and found that the numbers of WT and CD147-deficient Tregs were comparable (Fig. 1H). Importantly, we further observed that the average level of Foxp3 expression in CD147^T-KO^ Tregs was decreased, and more CD147^T-KO^ Tregs lost their Foxp3 expression completely (Fig. 1I). Taken together, these data suggest that CD147 is indispensable for supporting Tregs and their immunosuppressive function in vivo.

### Loss of CD147 expression accelerates Foxp3 protein degradation

**Fig. 2:**
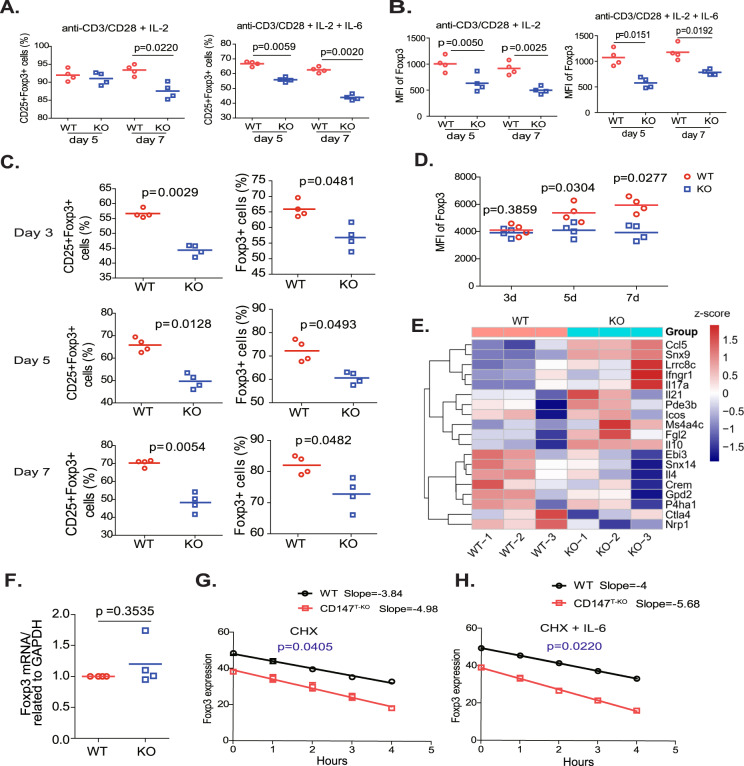
Loss of CD147 expression accelerates Foxp3 protein degradation. **A, B** Sorted CD147-sufficient or CD147-deficient Tregs from the LNs and spleens of wild-type and CD147^T-KO^ mice were labeled with CellTrace proliferation dye and stimulated with anti-CD3/anti-CD28-coated microbeads and 50 IU/ml IL-2 with or without 50 ng/ml IL-6 for 5 or 7 days. CD25 and Foxp3 expression were detected by flow cytometry. **A** Statistical analysis of CD25 and Foxp3 expression. **B** Statistical analysis of Foxp3 MFI. **C–D** CD147-sufficient or CD147-deficient naïve CD4 + T cells were sorted from the spleens of wild-type or CD147^T-KO^ mice and then stimulated with anti-CD3/28-coated microbeads, 25 IU/ml IL-2, 10 µg/ml anti-IL6 and 10 ng/ml TGF-β for 3, 5 or 7 days. **C** Statistical analysis of CD25 and Foxp3 expression as measured by flow cytometry. **D** Statistical analysis of Foxp3 MFI. **E–H** CD147-sufficient or CD147-deficient naïve CD4 + T cells were sorted from the spleens of wild-type or CD147^T-KO^ mice and then stimulated with anti-CD3/28-coated microbeads, 25 IU/ml IL-2, 10 µg/ml anti-IL6 and 10 ng/ml TGF-β for 5 days. **E** RNA-seq analysis. A heatmap of differentially expressed genes. The columns represent samples, and the rows represent genes. Gene expression levels in the heat maps are *z* score. **F** Detection of Foxp3 mRNA expression by qPCR using total RNA from iTregs. **G** CHX (15 µg/ml) was added to the iTreg culture system on Day 5, and Foxp3 expression was quantified by flow cytometry after 1, 2, 3, and 4 h, as indicated. **H** CHX (15 µg/ml) and IL-6 (50 ng/ml) were added to the iTreg culture system on Day 5, and Foxp3 expression was detected by flow cytometry after 1, 2, 3, and 4 h, as indicated

To determine how CD147 supports the stability of mature Tregs, we established an in vitro assay to mimic the in vivo inflammatory environment. To this end, CD147-sufficient or CD147-deficient Tregs were stimulated with anti-CD3/28 and IL-2 in the absence or presence of IL-6, a well-known inflammatory cytokine that destabilizes mature Tregs [10]. We observed that TCR (T cell receptor) plus IL-6 signaling led to a reduction in Treg numbers, and CD147 deficiency accelerated this loss (Figs. 2A & S2A). This CD147 deletion-associated Treg loss was not due to defects in survival or proliferation (Fig. S2B & C) but was most likely caused by severely decreased Foxp3 expression (Fig. 2B). Reduced Foxp3 expression was also observed during inducible Treg (iTreg) differentiation, regardless of the number of Foxp3+ cells or the level of Foxp3 expression in individual cells (Figs. 2C, D & S2D). However, when these differentiated cells were subjected to RNA-seq analysis, we found that although many downstream effector molecules were regulated by Foxp3, either directly (CTLA-4 and others) or indirectly (IL-10, EBI3, etc.), they were consistently suppressed due to CD147 deletion [30], and the mRNA expression of Foxp3 itself remained unchanged (Fig. 2E). Moreover, quantitative PCR analysis of independent iTreg samples confirmed no significant alteration in Foxp3 expression at the mRNA level (Fig. 2F). Therefore, this posttranscriptional regulatory mechanism appears to be evolutionally conserved and to regulate iTreg differentiation in murine CD4 + T cells.

These findings suggest that CD147 regulates Foxp3 levels at the translational or posttranslational level. To assess these possibilities, we applied cycloheximide (CHX), a general inhibitor of protein translation, to iTreg cells. Then, with its synthesis halted, we monitored the time-dependent decay of the Foxp3 protein. Foxp3 turnover was observed to occur in WT Treg cells but was significantly accelerated in CD147-deficient Tregs (Fig. 2G). Moreover, upon IL-6 administration, Foxp3 turnover was more robust in WT Tregs, but markedly accelerated decay was still observed in CD147-deficient Tregs (Fig. 2H). Based on these results, we concluded that CD147 is involved in maintaining Foxp3 protein stability.

### CD147-dependent Foxp3 stabilization requires a trans-acting ligand

**Fig. 3:**
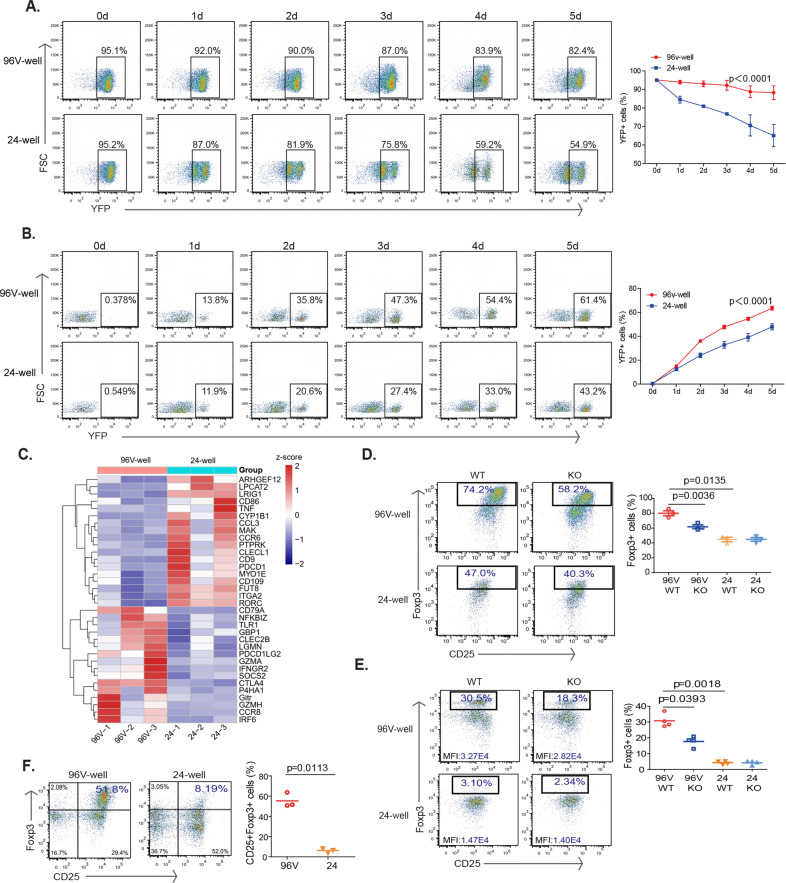
CD147-dependent Foxp3 stabilization requires a trans-acting ligand. **A, B** YFP + cells and naïve CD4 + cells were sorted from the LNs and spleens of Foxp3-YFP mice. **A** YFP + cells were seeded in V-shaped 96-well plates or flat 24-well plates and incubated with anti-CD3/anti-CD28-coated microbeads and 50 IU/ml IL-2. Flow cytometry analysis the YFP + cells. **B** Naïve CD4 + cells were seeded in V-shaped 96-well plates or flat 24-well plates and incubated with anti-CD3/28-coated microbeads, 25 IU/ml IL-2, 10 µg/ml anti-IL6 and 10 ng/ml TGF-β. Flow cytometry analysis the YFP + cells. **C** Naïve CD4 + cells were seeded in V-shaped 96-well plates or flat 24-well plates and incubated with anti-CD3/28-coated microbeads, 25 IU/ml IL-2, 10 µg/ml anti-IL6 and 10 ng/ml TGF-β. Five days later, the same number of cells in the two wells were collected for RNA-seq analysis. A heatmap of differentially expressed genes. The columns represent samples, and the rows represent genes. Gene expression levels in the heat maps are *z* score. **D** CD4 + CD25 + cells were seeded V-shaped 96-well plates or flat 24-well plates and incubated with anti-CD3/anti-CD28-coated microbeads and 50 IU/ml IL-2. Flow cytometry analysis of Foxp3+ cells. **E** Naïve CD4 + cells were seeded in V-shaped 96-well plates or flat 24-well plates and incubated with anti-CD3/28-coated microbeads, 25 IU/ml IL-2, 10 µg/ml anti-IL6 and 10 ng/10 ng/ml TGF-β. Flow cytometry analysis of Foxp3+ cells. **F** Naïve CD4 + T cells were sorted from human peripheral blood and then seeded in V-shaped 96-well plates or flat 24-well plates and incubated with anti-CD3/28-coated microbeads, 25 IU/ml IL-2, 10 µg/ml anti-IL6 and 10 ng/ml TGF-β. For all experiments, 2 × 10^5^ cells were cultured in V-shaped 96-well plates to allow sufficient contact, 5 × 10^4^ cells were cultured in flat 24-well plates to allow limited contact, and Foxp3 levels were detected by flow cytometry

As a membrane receptor, CD147 can be engaged by either cis-acting ligands, such as monocarboxylic acid transporters [31], GLUT1 [32], and CD44 [33] expressed on the same cells, or by trans-acting ligands, such as rhoptry-associated proteins [20], S100A9 [34], and cyclophilins [35] expressed on neighboring cells. To distinguish between these two possibilities, we manipulated cell-to-cell interactions during Treg culture or iTreg differentiation. Briefly, we seeded either 2 × 10^5^ cells into V-shaped 96-well plates to ensure sufficient contact or 5 × 10^4^ cells into flat 24-well plates to limit contact (Fig. S3A). First, we isolated YFP + cells from Foxp3-YFP mice, and after 5 days of culture with anti-CD3/28 stimulation, significantly more YFP + Treg cells lost YFP expression in 24-well, flat culture plates than in 96-well, V-shaped culture plates (Fig. 3A). Similarly, under conditions favorable to iTreg generation, naïve CD4 + T cells exhibited reduced YFP expression in 24-well, flat culture plates (Fig. 3B). RNA-seq analysis revealed that the expression of some Foxp3-regulated genes, such as CTLA4, GITR, and CD79a, was downregulated in the 24-well, flat culture plates (Fig. 3C). Moreover, we collected CD4 + CD25 + cells and seeded either 2 × 10^5^ cells into V-shaped 96-well plates or 5 × 10^4^ cells into flat 24-well plates. Upon comparing the stability of WT versus CD147-deleted Tregs in 24-well, flat culture plates, we found that CD147 deficiency failed to induce more severe loss of Foxp3 expression (Fig. 3D). Similarly, under the same conditions favorable to iTreg generation, the differences in Foxp3 expression between WT and CD147-deleted T cells were also decreased in 24-well, flat culture plates (Fig. 3E). This requirement for cell-to-cell interactions during iTreg differentiation was recapitulated in human CD4 + T cells (Fig. 3F). Taken together, these results suggest that cell-to-cell contact, hence a trans-acting ligand, is necessary for the CD147-mediated maintenance of Foxp3 stability.

### CD98 is the ligand that engages CD147 to induce Foxp3 protein expression

**Fig. 4:**
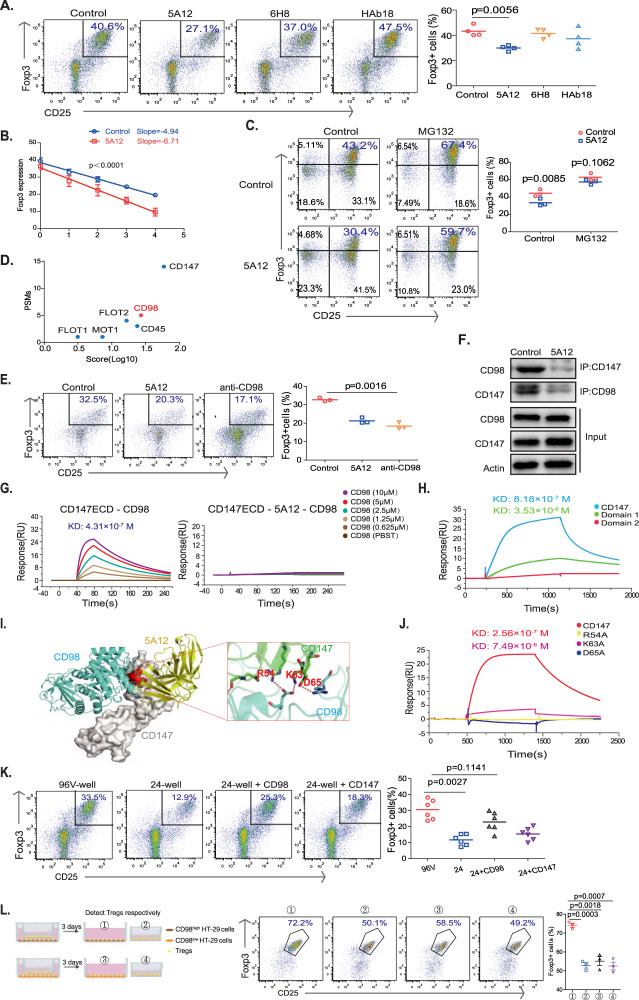
CD98 is the ligand that engages CD147 to induce Foxp3 protein expression. **A–C** A total of 2 × 10^5^ human naïve CD4 + cells were seeded in V-shaped 96-well plates and incubated with anti-CD3/28-coated microbeads, 25 IU/ml IL-2, 10 µg/ml anti-IL6 and 10 ng/ml TGF-β. **A** HAb18G, 5A12, or 6H8 antibodies (4 µg/ml) were added to assess their ability to block iTreg differentiation, as indicated. Foxp3 expression levels were detected by flow cytometry. **B** CHX (15 µg/ml) was added to the iTreg differentiation system with or without 4 µg/ml 5A12, and the levels of Foxp3 were detected by flow cytometry. The slope of the line represents the rate of Foxp3 degradation. **C** MG-132 (10 µM) was added to the iTreg culture system with or without 5A12, and the levels of Foxp3 were detected by flow cytometry. **D** Score (−10*Log(P), where P is the probability that the observed match is a random event) and peptide spectrum match (PSM) analysis of the six membrane proteins identified in the mass spectrometry results. **E** A total of 2 × 10^5^ naïve CD4 + cells were seeded in V-shaped 96-well plates with anti-CD3/28-coated microbeads, 25 IU/ml IL-2, 10 µg/ml anti-IL6 and 10 ng/ml TGF-β, after which 4 µg/ml 5A12 or anti-CD98 antibodies were added to block iTreg differentiation, as indicated. Levels of Foxp3 expression were detected by flow cytometry. **F** The interaction between CD147 and CD98 was detected by coimmunoprecipitation and western blotting. **G** Interaction between the CD147 extracellular section (ECD) and CD98 ECD was detected by SPR. **H** SPR experiments were performed to detect the interaction between CD98 and domain 1 or domain 2 of CD147. **I** Structure of the CD147 and 5A12 complex. ClusPro (https://cluspro.bu.edu/home.php) was used to predict the most likely site of CD147-CD98 binding. The dotted line represents the salt bridge. **J** SPR experiments with CD98 and domain 1 of CD147 with the indicated point mutations. **K** Human naïve CD4 + cells were seeded into V-shaped 96-well plates or flat 24-well plates and incubated with anti-CD3/28-coated microbeads, 25 IU/ml IL-2, 10 µg/ml anti-IL6 and 10 ng/ml TGF-β for 5 days. A total of 2 × 10^5^ cells were cultured in V-shaped 96-well plates, and 5 × 10^4^ cells were cultured in flat 24-well plates. Recombinant CD147 or CD98 proteins (8 µg/ml) were added to the 24-well plates, as indicated. Numbers of Foxp3+ cells were detected by flow cytometry. The data are shown as the mean ± SEM. **L** CD4 + CD25 + Tregs were cocultured with the CD98^high^ or CD98^low^ colon cancer cell line HT-29. Some of the Tregs were in contact with HT-29 cells, while others were separated and did not contact each other. Three days later, Tregs were collected for flow cytometry analysis.

Many proteins, including CD147 itself (through homotypic binding) [36], have been identified as ligands that engage the extracellular domains of CD147 and induce various activities [15]. We decided to determine the ligand associated with Foxp3 stability using a structural-functional approach. To this end, our laboratory has developed a series of functional monoclonal antibodies that target different structural domains of human CD147, including HAb18, 6H8, and 5A12 antibodies [20, 37, 38]. We thus set up an iTreg induction assay to test whether any of these antibodies interfered with the binding of the unknown ligand to suppress iTreg differentiation. In this way, we found that the monoclonal antibody 5A12 suppressed Foxp3 expression (Fig. 4A) in a dose-dependent manner (Fig. S3B). Upon translational inhibition using CHX, similar to the phenotype of genetic CD147 depletion in mouse Treg cells, 5A12-induced blockade also accelerated Foxp3 degradation in human Tregs (Figs. 4B & S3C). Furthermore, when the panproteasomal inhibitor MG-132 was applied to iTreg cultures, the Foxp3 protein was protected from degradation in the presence of 5A12 antibody (Fig. 4C). These results collectively suggest that 5A12 antagonizes ligand binding to trigger Foxp3 destabilization.

This antagonizing feature of 5A12 allowed us to perform “subtraction proteomics” to identify the CD147 ligand relevant for Foxp3 stability. Using cell lysates from in vitro induced iTregs, we assessed the flow through of three cell lysates through a spin column conjugated with 6H8 antibody. The first group was the whole iTreg lysate (whole lysate) used to elute the complex of CD147 and its ligands; the second group was the whole lysate plus 6H8 (nonspecific binding control) used to elute nonspecific proteins that bind to 6H8; the third group was the whole lysate plus 5A12 (subtraction group) used to elute the complex of CD147 and its ligands except those blocked by 5A12 (Fig. S3D). Therefore, CD147 ligands blocked by 5A12 were subtracted from the whole lysate with the nonspecific binding control and the subtraction group (Fig. S3E). In this remaining fraction, 32 proteins, including 6 membrane proteins and 26 intracellular proteins, were identified in three independent experiments. Excluding CD147 itself, the CD98 glycoprotein [39] had the highest score and the highest occurrence frequency (Figs. 4D & S3F). Therefore, our subsequent experiments focused on CD98.

Similar to 5A12, antibody blocking of CD98 exerted the same suppressive effect on iTreg differentiation (Fig. 4E), consistent with the hypothesis that CD98 is functionally associated with CD147. To validate our mass spectrometry data, we performed Co-IP experiments using recombinant CD147 and CD98 proteins (Fig. 4F). While CD98 was pulled down when CD147 was used as bait, and vice versa, the addition of the 5A12 antibody disrupted this interaction (Fig. 4F). Using surface plasmon resonance (SPR), the binding affinity of this interaction was determined to have a KD of 431 nM. Moreover, SPR revealed that the CD98-CD147 interaction could be completely abolished by the addition of the 5A12 antibody (Fig. 4G). This inhibition of binding is ligand-specific because the CD147-caveolin interaction remained intact in the presence of 5A12 (Fig. S3G).

We further dissected the molecular interaction between CD98 and CD147. The extracellular portion of CD147 (BSG1) is composed of two IgG fold domains termed domain 1 and domain 2. When these two domains were folded in vitro and applied to SPR, no interaction was detected between domain 2 and CD98, whereas domain 1 bound CD98 with a reduced affinity (K_D_ = 3.53 μM; Fig. 4H). Computational simulation predicted that the amino acids R54, K63, and D65 constitute the docking surface of CD98 on CD147. CD147 mainly binds to heavy chains of 5A12, including amino acids 61-66 (VLKEDA). Therefore, 5A12 blocked the binding of CD147 and CD98 (Fig. 4I). This prediction was validated by SPR: the K63A mutation in CD147 severely reduced its binding affinity to CD98, while the R54A and D65A mutations completely abolished the interaction (Fig. 4J).

Because CD147 and CD98 function as both ligands and receptors, we set out to determine which molecule carries out downstream signaling in Treg cells. To this end, we added recombinant proteins to 24-well culture plates in which iTreg induction was carried out with limited cell-to-cell contact. While the CD147 protein failed to rescue Foxp3 expression under these conditions, recombinant CD98 partially stabilized Foxp3 expression (Fig. 4K), strongly suggesting that CD147 functions as a receptor to mediate CD98 signaling in Treg cells. To further verify the role of CD98, CD4 + CD25 + Tregs were cocultured with the CD98^high^ or CD98^low^ colon cancer cell line HT-29 (Fig. S3H). Some of the Tregs were in contact with the HT-29 cells, while others were separated and did not contact each other (Fig. 4L). The results showed that Tregs in contact with CD98^high^ HT-29 cells maintained Foxp3 levels at the highest intensity (Fig. 4L), further indicating the importance of CD98 in the environment for maintaining Foxp3 stability in Tregs.

### CD147 stabilizes Foxp3 by sequestering CDK2 from activation

**Fig. 5:**
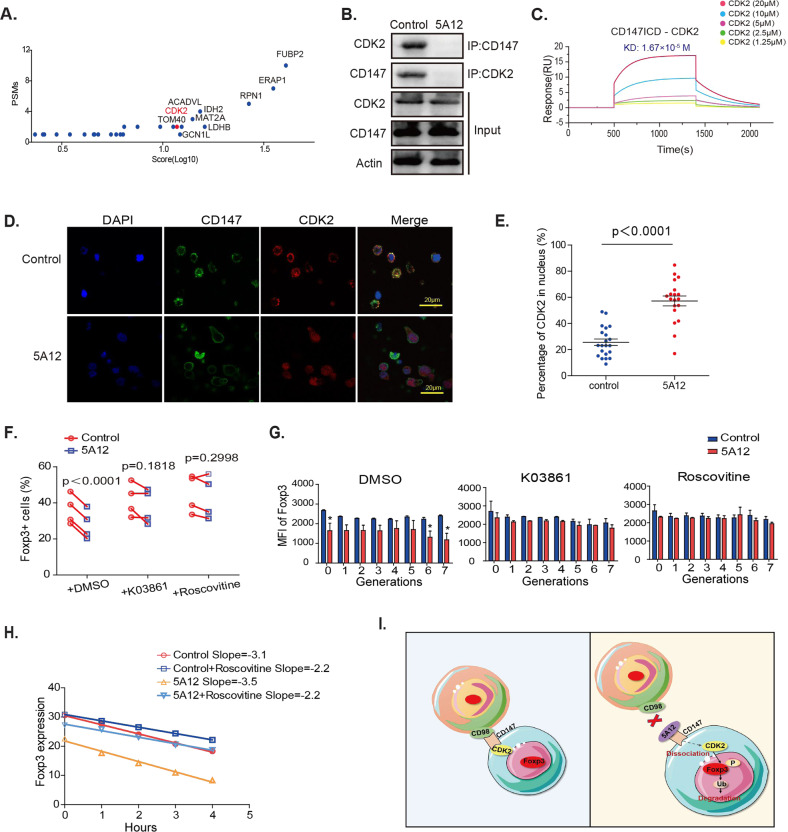
CD147 stabilizes Foxp3 by sequestering CDK2. **A** PSMs and score (log10) analysis of 37 intracellular proteins detected by mass spectrometry. **B** The interaction between CD147 and CDK2 was detected by coimmunoprecipitation assay and western blotting. **C** The interaction between CD147’s intracellular domain (ICD) and CDK2 was measured by SPR. **D** Tregs were cultured in V-shaped 96-well plates with or without 5A12 and fixed. Confocal experiments were performed to visualize CDK2 (red) and CD147 (green) expression. **E** Statistical analysis of the percentage of cells expressing nuclear CDK2 in the control and 5A12 groups. Each point represents an image, and the images in each group were taken from three wells. **F** Naïve CD4 + T cells (2 × 105) were incubated with CFSE and then seeded in V-shaped 96-well plates and incubated with anti-CD3/28-coated microbeads, 25 IU/ml IL-2, 10 µg/ml anti-IL6 and 10 ng/ml TGF-β with or without 5A12 antibody for 5 days. The indicated CDK2 inhibitors were then added to the culture system. Cell proliferation and Foxp3 expression were measured by flow cytometry. **G** Analysis of Foxp3 MFI (*p* < 0.05). **H** CHX (15 µg/ml) was added to the iTreg culture system with or without CDK2 inhibitors (2 µM roscovitine or 0.5 µM K03861), as indicated. Foxp3 levels were assessed by flow cytometry. **I** Model of CD147-mediated Foxp3 stabilization.

To identify signaling modules downstream of CD147, we analyzed the intracellular pool of 26 proteins co-IPed with CD147 (Fig. S4A), focusing on the top ten proteins (Fig. 5A). STRING online analysis of the protein–protein interactions showed that CDK2 has a relationship with Foxp3 (Fig. S4B). Therefore, we hypothesized that CD147’s intracellular segments might regulate Foxp3 stability by interacting with CDK2. Indeed, Co-IP assays using recombinant proteins detected a specific interaction between CD147’s intracellular domain and CDK2 (Fig. 5B). Using SPR, we determined that CD147’s intracellular segment binds to CDK2 with a KD of 16.7 μM (Fig. 5C). We next sorted human Tregs and cultured them in 96-well, V-shaped plates. We imaged CDK2 localization in response to this forced contact and in the presence or absence of 5A12 antibody. Confocal microscopic imaging revealed that cell-to-cell contact allowed ~74% of CDK2 to be colocalized with CD147 molecules in the proximity of the cell membrane; in contrast, after blocking 5A12, CDK2 dissociated from CD147 and the membrane region such that ~57% of the protein was detected in the cell nuclei (Fig. 5D, E).

We next assessed whether CDK2 kinase activity is required for CD147-mediated Foxp3 stabilization. To this end, we individually applied two CDK2 inhibitors, K03861 (a specific inhibitor) and roscovitine (an inhibitor of CDK2, CDK5, and Cdc2), to iTreg differentiation cultures. While these inhibitors did not inhibit cell cycle progression or T cell proliferation, both rescued 5A12-inhibited iTreg differentiation (Figs. 5F & S4C). This rescue was associated with enhanced Foxp3 expression at each individual round of cell division during differentiation (Fig. 5G). Mechanistically, CHX-based protein decay analysis revealed that CDK2 inhibition reversed the 5A12-induced Foxp3 protein reduction (Figs. 5H & S4D). Taken together, we propose that CD98 engagement allows CD147 to recruit and sequester CDK2 such that disrupting this engagement releases CDK2 to the nucleus, allowing it to phosphorylate Foxp3 for degradation (Fig. 5I).

### The optimal distribution of Foxp3 + Tregs depends on CD98 expression under both pathological and physiological conditions

**Fig. 6:**
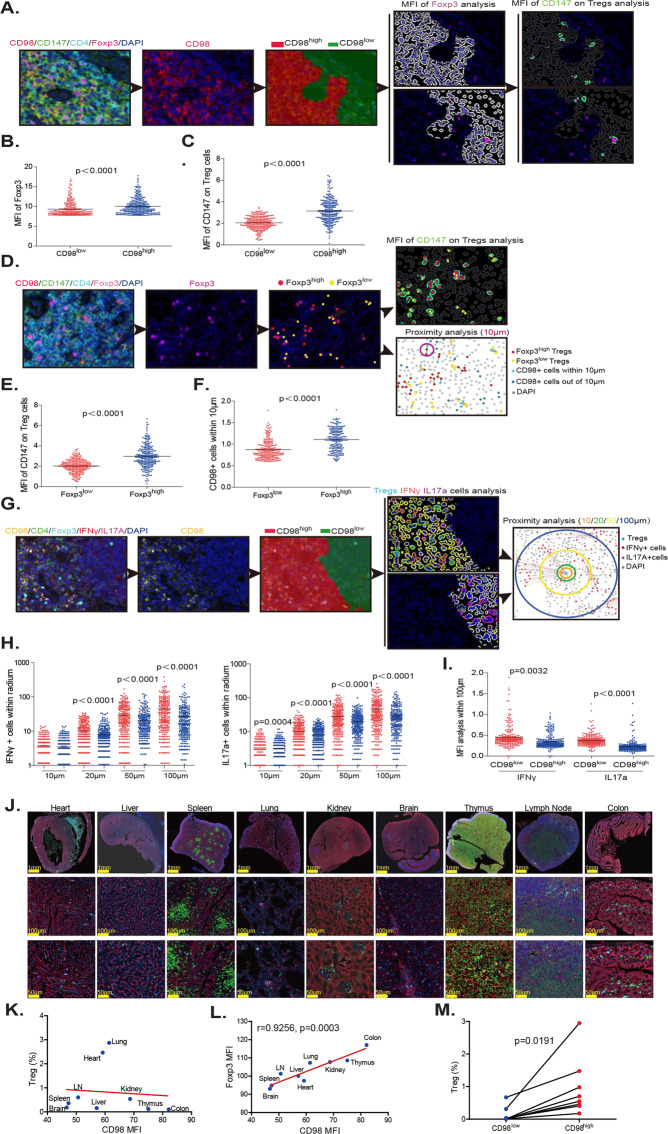
The optimal distribution of Foxp3 + Tregs depends on CD98 expression under both pathological and physiological conditions. The intestinal tissue sections of 26 patients with IBD, including 20 patients with ulcerative colitis and 6 patients with Crohn’s disease, were stained with antibodies recognizing CD98, CD147, CD4, Foxp3, IFNγ, and IL-17 and analyzed by multicolor immunofluorescence labeling. Each point in a scatter plot represents the average of ten similar values. **A** Strategy for analyzing Foxp3 expression and the average fluorescence intensity of CD147 on Tregs in CD98^high^ and CD98^low^ regions. **B** Foxp3 expression in CD98^high^ and CD98^low^ regions. **C** Average fluorescence intensity of CD147 expression on Tregs in the CD98^high^ and CD98^low^ regions. **D** Strategy for analyzing the average fluorescence intensity of CD147 expression on Foxp3^high^ and Foxp3^low^ cells and the number of CD98 + cells within 10 μm of Foxp3^high^ and Foxp3^low^ cells. **E** Average fluorescence intensity of CD147 expression on Foxp3^high^ and Foxp3^low^ cells. **F** Number of CD98 + cells within 10 μm of Foxp3^high^ and Foxp3^low^ cells. **G** Strategy for analyzing the number of IFNγ + and IL17a + cells within 10, 20, 50, and 100 μm of Tregs in CD98^high^ and CD98^low^ regions. **H** Numbers of IFNγ + and IL17a + cells within 10, 20, 50, and 100 μm of Tregs in CD98^high^ and CD98^low^ regions. **I** Average fluorescence intensity of IFNγ + and IL17a + cells within 100 μm of Tregs in CD98^high^ and CD98^low^ regions. **J** The heart, liver, spleen, lung, kidney, lymph node, thymus, brain and colon from normal C57BL/6 mice were stained and analyzed by multiplex immunofluorescence for CD98, CD4 and Foxp3. These sections were scanned and analyzed by Vectra Polaris. **K** Correlation between the percentage of Tregs and the MFI of CD98 in each organ. **L** Correlation between the MFI of Foxp3 and CD98 in each organ. All correlations were analyzed by Pearson analysis. **M** Each organ was divided into CD98^high^ and CD98^low^ regions, and the percentage of Tregs in the two regions was compared.

To determine whether increased numbers of Tregs in IBD are related to high intestinal CD98 expression, we stained intestinal tissue sections of 26 patients with IBD, including 20 patients with ulcerative colitis and 6 patients with Crohn’s disease, for CD98, CD147, CD4, Foxp3, IFNγ, and IL-17 using multicolor immunofluorescence labeling. To this end, we divided slides into CD98^high^ and CD98^low^ regions and analyzed the fluorescence intensity of Foxp3 and CD147 expression in Tregs in these two regions (Figs. 6A & S5A). This revealed that Foxp3 expression in the CD98^high^ region was higher than that in the CD98^low^ region, as was the average fluorescence intensity of CD147 expression on Tregs (Fig. 6B, C). This indicates that Foxp3 expression is positively correlated with CD98 expression in the environment. Then, we divided Treg cells into Foxp3^high^ and Foxp3^low^ Treg cells and analyzed CD147 expression on Tregs and the number of CD98 + cells within 10 μm regions surrounding each Treg (Figs. 6D & S5B). The results demonstrated that the expression of CD147 was significantly stronger in Foxp3^high^ Tregs (Fig. 6E); moreover, the number of CD98 + cells near Foxp3^high^ Tregs was higher (Fig. 6F), as was the CD98 fluorescence intensity (Fig. S5C). These data suggest that CD147 determines the expression of Foxp3 and may interact with CD98 + cells. Finally, we analyzed the expression of IFNγ and IL17a around Tregs in the CD98^high^ and CD98^low^ regions (Fig. 6G) and found that the number (Fig. 6H) and fluorescence intensity (Fig. 6I) of IFNγ + and IL17a + cells within 100 μm of Tregs in the CD98^high^ region were significantly reduced, indicating that Tregs in the CD98^high^ region have stronger suppressive functionality. Based on these results, we concluded that high levels of CD98 stabilize Tregs under pathological IBD conditions.

The heart, liver, spleen, lung, kidney, lymph node, thymus, brain and colon from normal C57BL/6 mice were stained and analyzed by multiplex immunofluorescence to observe whether the distribution of Foxp3 Tregs was dependent on CD98 expression under normal physiological conditions (Fig. 6J). The results showed that although the number of Tregs was not consistent with the expression of CD98 (Fig. 6K), the expression intensity of Foxp3 was highly consistent with that of CD98 (Fig. 6L). In the same organ, Foxp3+ Tregs tended to be distributed in CD98^high^ regions (Fig. 6M). These results suggest that the optimal distribution of Foxp3+ Tregs depends on CD98 expression under physiological conditions.

### CD147^high^ iTregs successfully suppress the inflammatory response in CD4 T cell-driven experimental IBD in humanized mice

**Fig. 7:**
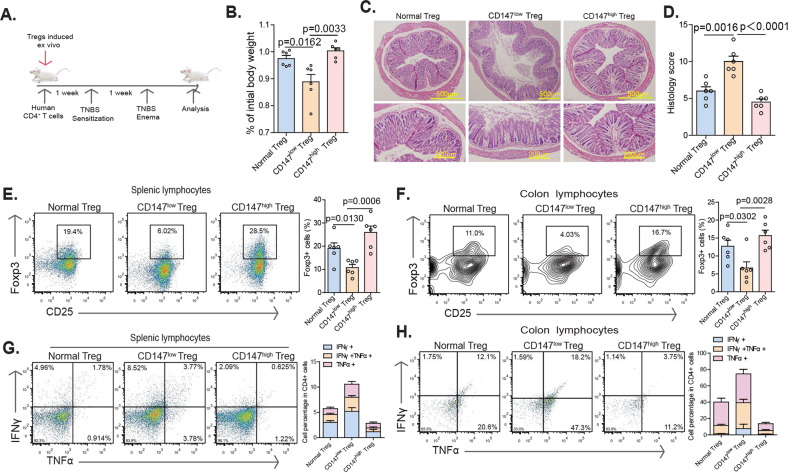
CD147^high^ Tregs successfully suppress T cell-driven experimental colitis in humanized mice. **A** Schematic representation of the workflow for the establishment of a TNBS-induced colitis model in humanized mice. **B** Weight changes in recipient mice after adoptive transfer normalized to their initial body weights before transfer. **C–D** Mice were euthanized on Day 5 after enema, and colon tissues were isolated for histopathological analysis. **C** Representative images of colon tissues stained with H&E. **D** Summary of histopathological scores. **E** Quantification of Tregs in spleen. **F** Quantification of Tregs in colon. **G** Splenic CD4 + T cells were stimulated with PdBU and ionomycin for 4 h and then stained for the intracellular cytokines IFNγ and TNFα. **H** Colon CD4 + T cells were stimulated with PdBU and ionomycin for 4 h and then stained for the intracellular cytokines IFNγ and TNFα.

To further investigate the effects of CD147 on human T cells in vivo, we established a 2,4,6-trinitrobenzene sulfonic acid (TNBS)-induced model of colitis in humanized mice. Here, 6–8-week-old NOG (NOD/Shi-scid/IL-2Rγnull) mice were divided into three groups, which were intraperitoneally injected with 2 × 10^6^ human CD4 + T cells mixed with 1 × 10^6^ iTregs, CD147^low^ iTregs or CD147^high^ iTergs. Then, 1 week after engraftment of each of these cell populations, the mice were sensitized to TNBS, and two weeks later, they were administered a single TNBS rectal challenge (Fig. 7A). The results of this experiment indicated that administration of CD147^high^ iTregs or normal iTregs to humanized mice followed by TNBS challenge provides protection against weight loss compared to administration of CD147^low^ iTregs (Fig. 7B). Moreover, histological evaluation of H&E-stained colon sections revealed a significant reduction in the severity of inflammation in mice receiving CD147^high^ iTregs or normal iTregs with a corresponding reduction in colitis score (Fig. 7C, D). Importantly, we observed a significant reduction in the percentage of Foxp3+ Tregs in the CD147^low^ group in both the spleen and colon (Fig. 7E, F), while the frequency of inflammatory cytokine production in these groups was significantly increased (Fig. 7G, H). Although the pathological characteristics of the CD147^high^ iTreg group were similar to those of the normal iTreg group, mice administered CD147^high^ iTregs had more Foxp3+ Tregs and fewer infiltrating inflammatory lymphocytes (Fig. 7E–H), making CD147^high^ Tregs more stable and more functional.

## Discussion

Given Tregs’ central role in immune regulation and peripheral tolerance, strict control of their suppressive function is required to establish a balanced immune response. Existing evidence suggests that the expression and activity of Foxp3 are regulated by posttranslational modification through the activity of intracellular factors, which is necessary for the proper regulation of Treg stability and activity [40, 41]. However, a critical area of Treg research that remains to be explored is the mechanisms by which extracellular signals regulate the expression, stability, and function of Foxp3. Here, we discovered that Tregs require cell-cell contact to stabilize the Foxp3 protein; this cell-cell contact includes Treg-Treg contact and Treg-other cell contact, which is achieved through the CD147-CD98 interaction. In IBD, CD98 is expressed on intestinal epithelial cells (IECs), Tregs and other infiltrating cells and continuously stimulates CD147 on Tregs to maintain Foxp3 stability. In an organism’s internal environment, CD98 is a widely expressed membrane protein, even on T cells [42], and can thus be considered a widespread environmental factor. Therefore, we speculated that after their development and formation, Tregs must be stabilized in a given environment by the presence of CD98.

The relationship between CD98 and Tregs or inflammatory responses has been reported in mice. High CD98 expression on mouse IECs results in imbalanced intestinal homeostasis and increased inflammatory responses induced by dextran sodium sulfate (DSS); thus, specific knockout of CD98 in IECs causes resistance to the DSS-induced enteritis response. This suggests that CD98 expression in IECs is not conducive to the formation of an immunosuppressive microenvironment [43]. However, this paper did not focus on other immune cell subsets, such as Tregs, so the relationship between CD98 expression in IECs and Treg activity could not be determined. In another report, heart transplantation into another mouse strain with T cell-specific CD98 depletion resulted in increased Treg numbers, leading to reduced alloantigen rejection [44]. Together, these data suggest that CD98, a proinflammatory factor, plays a critical role in controlling homeostatic and innate immune responses in the gut and GvHD. Although seemingly contrary to our study, these discrepancies are likely due to species differences: our conclusions are based on human Tregs. The most direct evidence concerning CD98 and Tregs reported in the literature is that mice lacking CD98 expression specifically in Foxp3+ Tregs exhibited decreased numbers of Tregs in the spleen through impaired isoleucine-induced activation of the mTORC1 pathway and an altered metabolic state. The CD98 heavy chain can form branched-chain amino acid transporter complexes by associating with several molecules, including Lat1, Lat2, y^+^Lat1, and y^+^Lat2, to maintain the proliferative state of Treg cells [45]. Therefore, another possibility was that CD98 and CD147 form an amino acid transport channel to maintain the stability of Treg cells; this hypothesis will require many experiments for verification. In addition, Tregs from Foxp3^Cre^CD98^flox/flox^ mice exhibit low CTLA-4 expression and defective suppressive activity, leading to lymphocyte infiltration into many tissues, including the pancreas, thyroid, stomach, liver, and salivary gland, of aged Foxp3^Cre^CD98^flox/flox^ mice [45]. These results are similar to ours, but the mechanistic explanation is different. In summary, current evidence indicates that regardless of whether CD98 is expressed in the intestine or on Tregs, it acts as an anti-inflammatory molecule to regulate Treg stability, either by stabilizing the Foxp3 protein or by maintaining Treg cell proliferation.

Our study further reveals that CD147-CD98 contact maintains Foxp3 protein stability through CDK2. In most cells, CDK2 acts as a cyclin kinase that binds cyclin E or cyclin A and then phosphorylates a series of downstream substrates, including Rb, CDC6, NPAF, P107, and others, to initiate DNA synthesis such that cells irreversibly enter S phase [46, 47]. However, in T cells, another cyclin-dependent kinase family member, CDK1, is necessary for cell cycle regulation [48, 49], whereas CD4 + T cells lacking CDK2 expression can proliferate normally [50]. Although CDK2 is not necessary for T cell proliferation, it promotes cytokine production in CD4 + T cells and inhibits Treg function [46]. Moreover, CDK2-deficient Tregs are more suppressive and ameliorate colitis in an in vivo mouse model of IBD; [50] this is primarily because the N-terminal amino acid sequence of Foxp3, which contains four CDK substrate motifs and is phosphorylated by CDK2, leading to Foxp3 instability [13]. Importantly, CDK2 can be regulated by extracellular factors. For example, although CDK2 is not necessary for T cell expansion in vitro, it responds to stimulation with a CD28 antibody, thereby improving T cell activity and expansion in vitro [51]. Therefore, in T cells, CDK2 is not a necessary cell cycle regulator but an environmental response molecule that regulates T cell differentiation and function [50, 52]. Our results show that CD98 interacts with CD147 on Tregs to suppress CDK2 activity and maintain Foxp3 stability. CDK2 must be located in the nucleus to function in cell cycle regulation; however, when CDK2 functions in T cell differentiation or functional regulation, it is located in the cytoplasm near the membrane. Our study reveals that CD147 is the key molecule that sequesters CDK2 from the nucleus to maintain Foxp3 stability.

## Supplementary information

Supplementary Data
