# Supplementary material for: CD98-induced CD147 signaling stabilizes the Foxp3 protein to maintain tissue homeostasis
Source: Cell Mol Immunol. 2021 Nov 10;18(12):2618–31. doi: 10.1038/s41423-021-00785-7 (PMC8632965; doi:10.1038/s41423-021-00785-7)
Supplement: Supplementary file 1 — Supplementary Data [file 41423_2021_785_MOESM1_ESM.pdf]

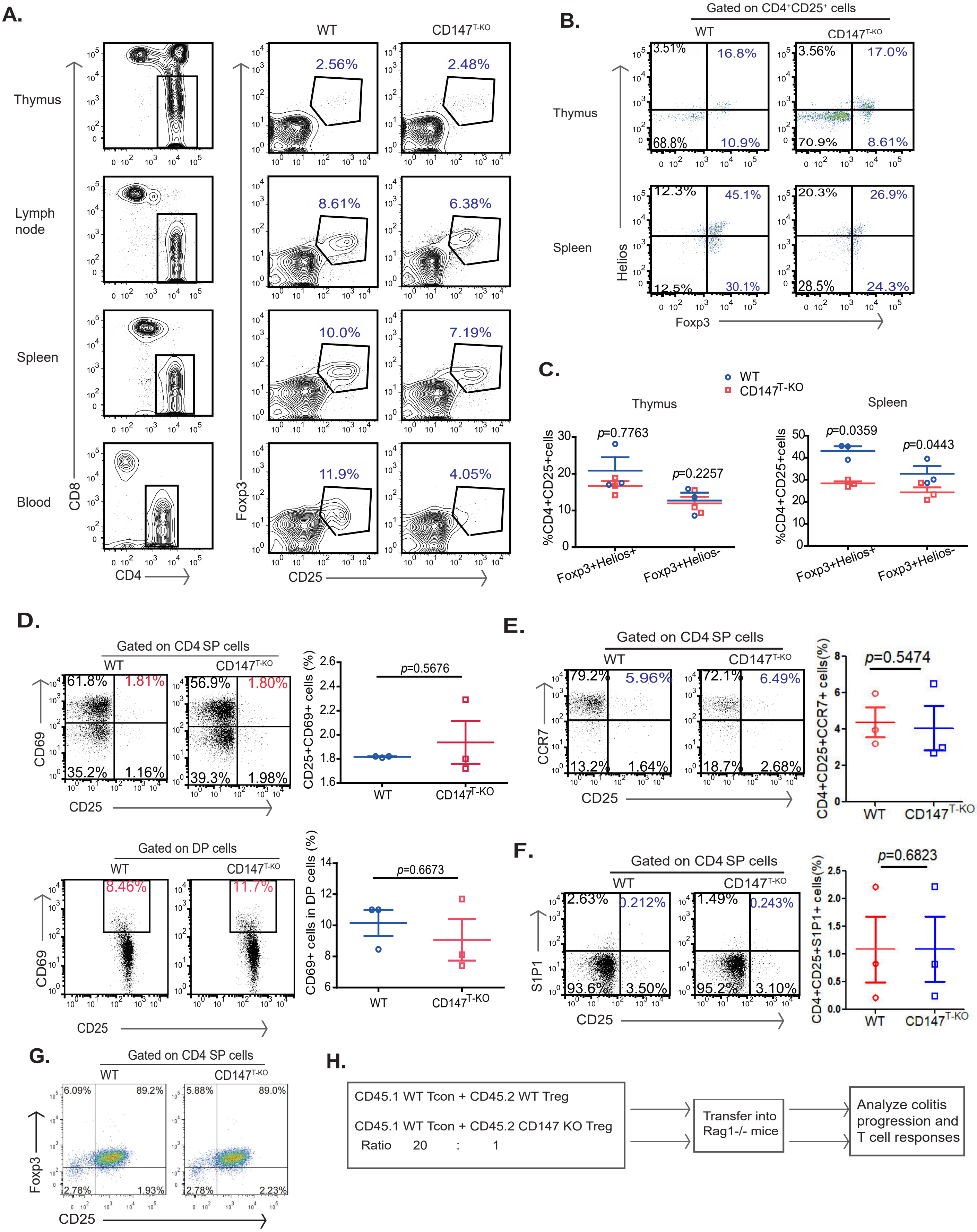

Figure S1. Loss of CD147 destabilizes Tregs in the periphery. A. Representative FACS plot of Foxp3+ Tregs in thymus, lymph nodes, spleen, and peripheral blood from wildtype and CD147$^{T\text{-}KO}$ mice. **B & C.** Foxp3 quantification and Helios analysis of thymic and splenic Tregs from wildtype and CD147$^{T\text{-}KO}$ mice. **D.** Detection of CD69 expression in DP cells and CD4+SP cells in both CD147$^{T\text{-}KO}$ and wildtype mice. **E.** CCR7expression in thymic Tregs from CD147$^{T\text{-}KO}$ and wildtype mice. **F.** S1P1R expression in thymic Tregs from CD147$^{T\text{-}KO}$ and wildtype mice. **G.** Detection of Foxp3 expression in CD45.2+CD4+CD25+ Tregs that were sorted from CD147T-KO mice or (WT) mice. **H.**Schematicrepresentation of workflow for the IBD model. Each point represents an individual mice.

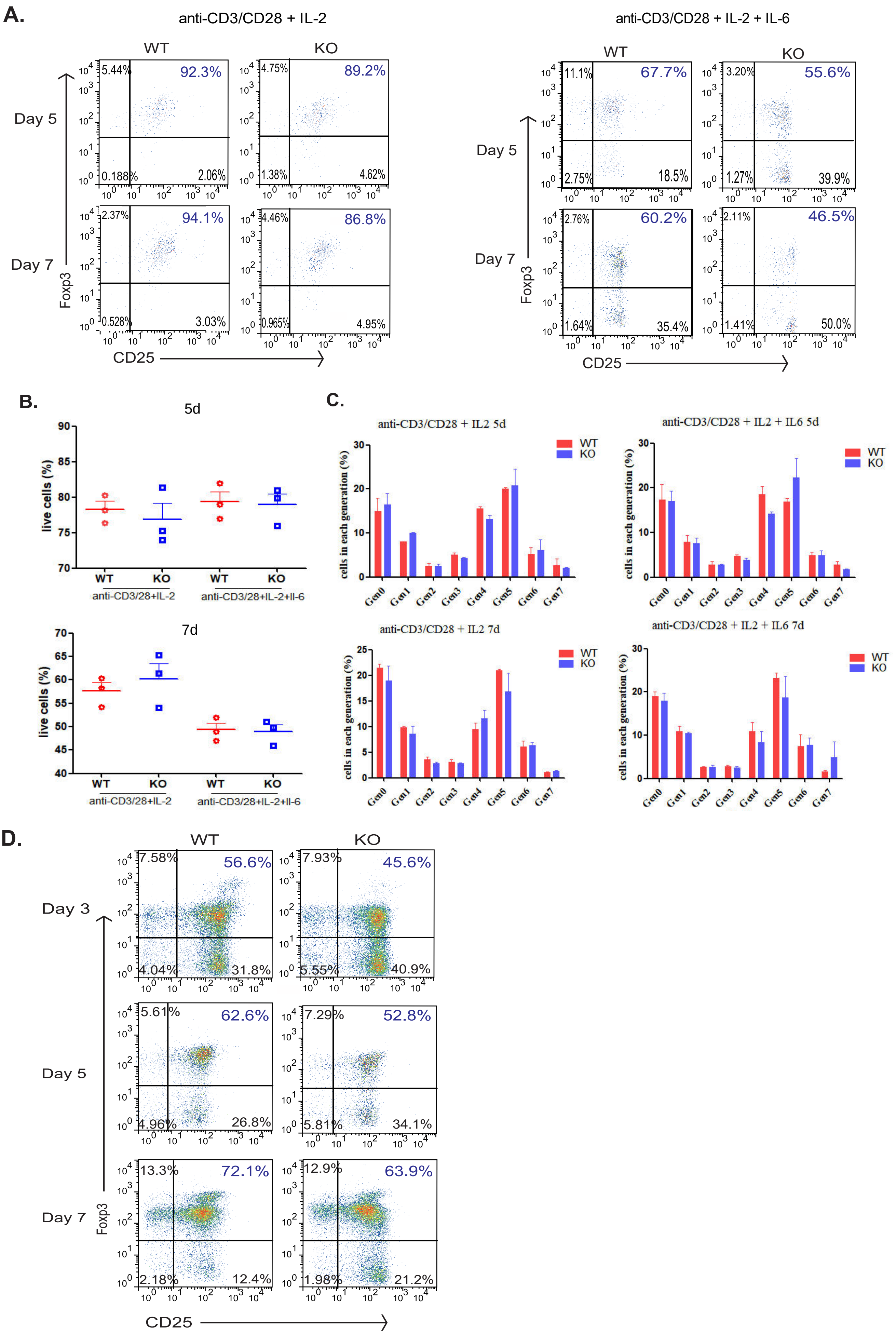

Figure S2. CD147 is required for the maintenance of Foxp3 protein stability in Tregs. CD147-sufficient or -deficientTregs were sorted from LNs and spleens of wildtype and CD147^{T-KO} mice, and then stimulated with anti-CD3/28 and IL-2 with or without IL-6. **A.** Representative FACS plots of CD25+Foxp3+ Tregs. **B.** Percentages of live cells at 5 and 7 days were determined by LIVE/DEAD Fixable VioletDead Cell Staining. **C.** Proliferation of live cells at 5 and 7 days was determined by dilution of CellTrace proliferation dye. **D.** CD147-sufficient or-deficient naïve CD4+ T cells were sorted from spleens of wildtype and CD147^{T-KO} mice, and then stimulated with anti-CD3/28,IL-2, andTGFβ for 3, 5 and 7 days. Representative FACS plots of CD25+Foxp3+ Tregs.

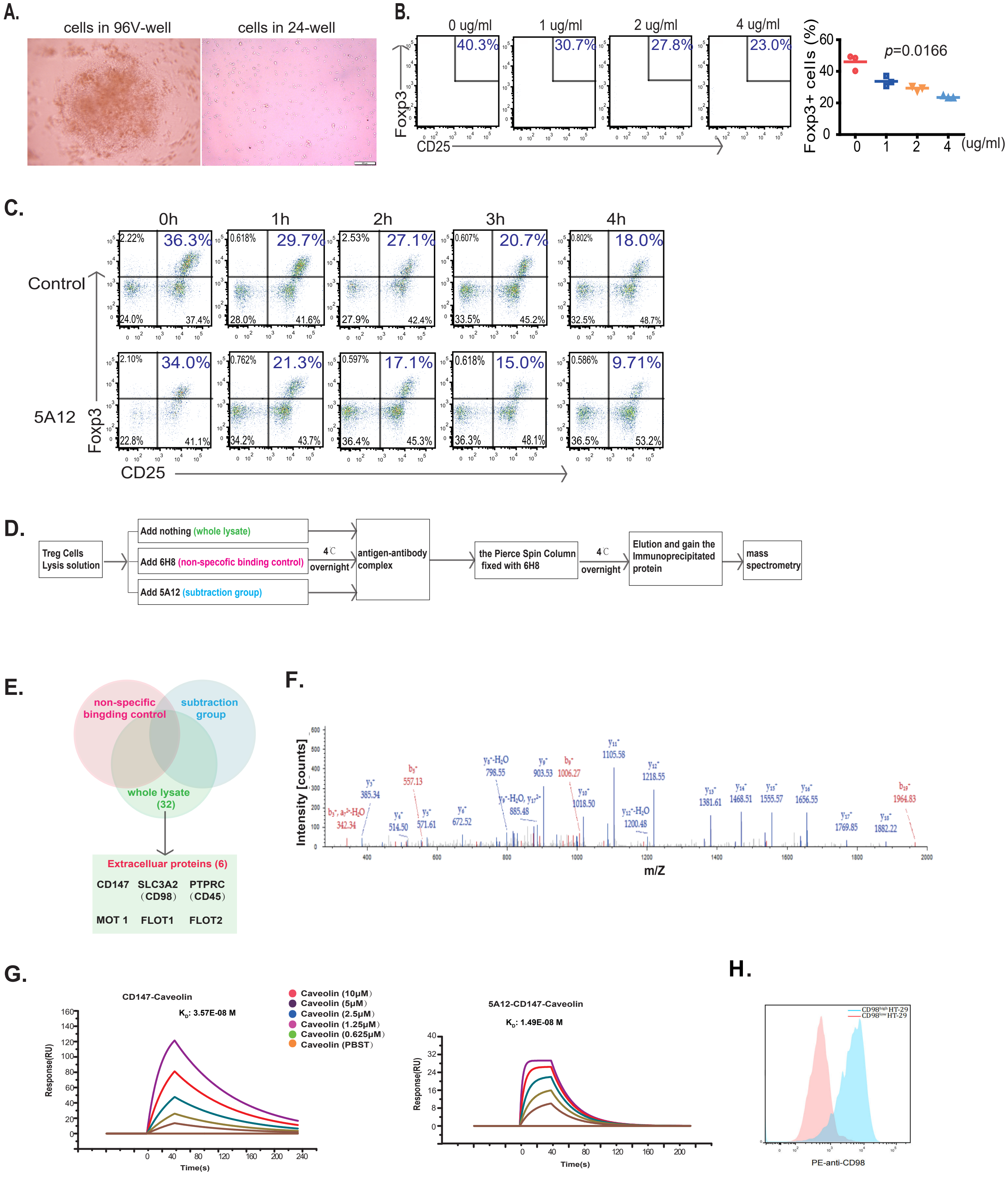

Figure S3. CD98 is the ligand engaging CD147 to enforce Foxp3 protein expression. A. Photographs of 5×10^{4} cells seeded in 24-well plates. **B.** 2×10^{5} human naïve CD4+ cells were seeded in 96V-well plates with anti-CD3/28, IL-2, and TGFβ, and increasing concentrations 5A12 antibodies were applied to block iTreg differentiation. Percentages of Foxp3+ cells were assessed by flow cytometry.**C.** CHX was added into iTreg cultures with or without 5A12, and Foxp3 levels were quantified by flow cytometry at the indicated time points. **D.** Schematic representation of the workflow for co-immunoprecipitation (CO-IP) experiments. **E.** Mass spectrometry analysis of co-immunoprecipitated proteins. **F.** CD98 detection by massspectrometry. **G.** SPR experiments using caveolin with CD147 with or without 5A12 antibody. **H.** Detection of CD98expression in CD98^{high} and CD98^{low} HT-29 cells by flow cytometry.

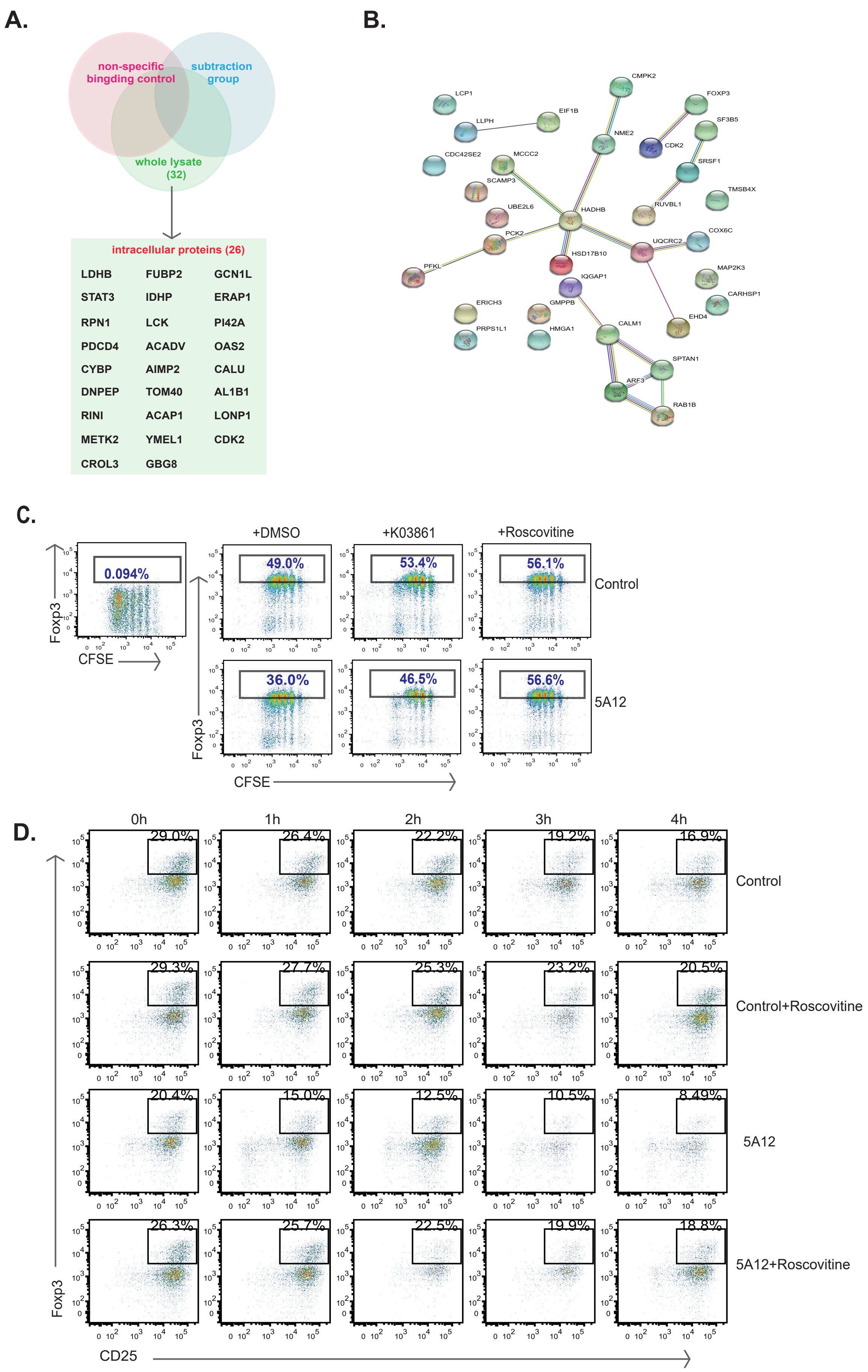

Figure S4. CD147 stabilizes Foxp3 by sequestering CDK2. A. 26 intracellular proteins identified by mass spectrometry. **B.**String online analysis of the relationship between these 26 proteins and Foxp3. **C.** 2×10^5 naïve CD4+ cells were incubated with CFSE andthen seeded in 96V-well plates with anti-CD3/28, IL-2, and TGFβ with or without 5A12 antibody for 5 days. The indicated CDK2 inhibitorswere then added to the culture system. Cell proliferation and Foxp3 levels were measured using flow cytometry.D. CHX was added to the iTreg culture system with or without the indicated CDK2 inhibitors. Foxp3 levels were assessed by flow cytometry.

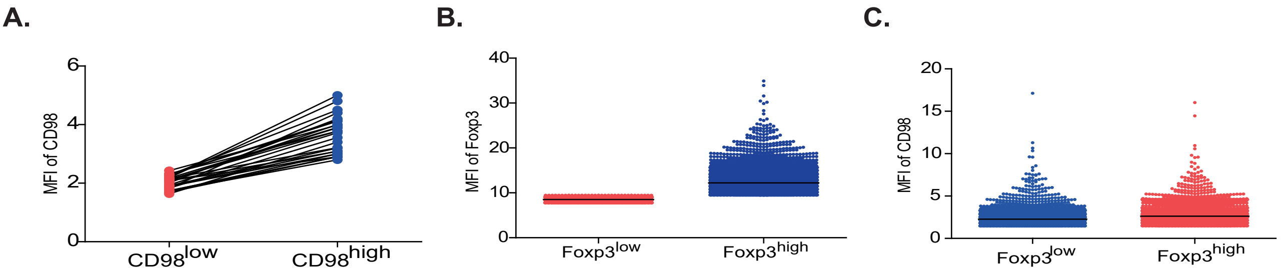

Figure S5. Intestinal CD98 interacts with CD147 on Tregs to maintain Foxp3 stability and Treg function in IBD. A. Average fluorescence intensity of CD98 in regions of CD98^{high} and CD98^{low}. B**.** Average fluorescence intensity of Foxp3 in Foxp3^{high} cells and Foxp3^{low} cells.C**.** Average fluorescence intensity of CD98+ cells within 10 μm around Foxp3^{high} cells and Foxp3^{low} cells.
